# A computational model of a strongly facilitating synapse

**DOI:** 10.1186/1471-2202-12-S1-P159

**Published:** 2011-07-18

**Authors:** Joanna Jędrzejewska-Szmek, Jarosław Żygierewicz, Aleksander Michalski

**Affiliations:** 1 Biomedical Physics, Faculty of Physics, University of Warsaw, ul.Hoża 69, 00-681 Warszawa, Poland; 2 Laboratory of Neurobiology of Development and Evolution, Nencki Institute of Experimental Biology, ul.L.Pasteura 3, 02-093 Warszawa, Poland

## 

We propose a new model of strongly facilitating synapse. It is described in terms of resources *R* which can be in two states: available and inactivated (recovery constant –*t_γ_* ). It assumes that for the release of neurotransmitter to the synaptic cleft a fraction (*u*) of available resources must bu used (as in [2]). This fraction is elevated by every AP (by a factor ~ u*U) and decays in between APs (facilitation constant – *t_f_* ). *u* related to the calcium concentration. It is further assumed that the activation of the neurotransmitter release machinery requires binding of 5 calcium ions to synaptotagmin[3], binding synaptic vesicles to the presynaptic membrane. Hence the postsynaptic current is proportional to u^5*^R*δ(t-t_AP_).

**Figure 1:**
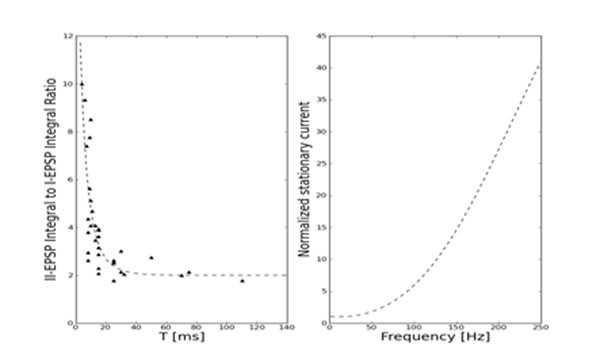
Best fit to the experimental results from [1] and stationary current predictions. Best-fit parameters can be found in Tab. 1.

The model allows to derive analytic formulas for the measures reported in the experimental literature, e.g. EPSP integrals [1] for consecutive action potentials arriving at the synapse. Those measures were used to estimate the model parameters so that it corresponds to the synapses reported in [1]. The obtained parameter values (Table 1) are in the physiologically plausible range. The best fit curve is presented in Fig. 1. The model allows to make predictions which can be used to validate it. In our case – the stationary current (normalized to the typical synaptic current) which can be seen in Fig. 1 – information coding is possible for physiological spike frequencies.

**Table 1:** Results of the models fit to the experimental data

parameter	*t_f_*	*U*	*t_r_*
Value and 68% confidence range in	10± 2 ms	0.18 ± 0.07	130 ms
